# FOXO3a Gene Polymorphism Associated with Asthma in Indian Population

**DOI:** 10.1155/2015/638515

**Published:** 2015-12-09

**Authors:** Shravani Barkund, Tejas Shah, Nikhil Ambatkar, Maithili Gadgil, Kalpana Joshi

**Affiliations:** Department of Biotechnology, Sinhgad College of Engineering, Vadgaon Budruk, Pune, Maharashtra 411041, India

## Abstract

Asthma is a chronic inflammatory disorder delineated by a heightened immunological response due to environmental or genetic factors. Single nucleotide polymorphism studies have shown that FOXO3a plays a pivotal role in maintaining immunoregulation. Polymorphism in FOXO3a has been linked to inflammatory diseases such as chronic obstructive pulmonary disease (COPD), Rheumatoid Arthritis, and Crohn's disease suggesting that FOXO3a may be associated with asthma. Airway inflammation in asthma is characterized by activation of T helper type 2 (Th2) T cells and Foxo family members are reported to play critical roles in the suppression of T cell activation. Thus this study was undertaken to investigate an association between single nucleotide polymorphism of the FOXO3a (rs13217795, C>T transition) gene and asthma in Indian population. To our knowledge we are the first ones reporting an association between FOXO3a and asthma.

## 1. Introduction

Asthma is a chronic immunological disorder characterized by inflammation of airways leading to obstruction, coughing, and wheezing in response to an allergen or inorganic pollutants [1]. Inflammation in asthma occurs due to excessive infiltration of cells predominantly T cells, eosinophils, neutrophils, macrophages, and mast cells along with elevated levels of cytokines such as IL-1, IL-4, IL-5, IL-6, IL-9, IL-10, IL-13, IL-17, and TNF-*α* [2–4]. The prevalence of asthma in India has increased to 15–20 million in the last 25 years and was found to be 1.69–3.47% mostly due to environmental factors in concert with genetic factors [1, 5, 6].

Forkhead (FOX) transcription factors play key roles in immunoregulation and homeostasis. FOXO is the subfamily of FOX which comprises four members FOXO1, FOXO3a, FOXO4, and FOXO6 [7]. The FOXO genes are the mammalian homologs of* Caenorhabditis elegans* DAF-16 which regulate a number of pathways such as insulin signalling, apoptosis, cell cycle transition, DNA repair, oxidative stress resistance, and longevity [8, 9]. Out of the four members of FOXO group only FOXO3a was found responsible for longevity in Chinese, Japanese, German, and Danish individuals [10]. FOXO3a is an integral component of protein kinase B/Akt pathway [11]. Akt protein is responsible for cell proliferation in the presence of growth factors thereby suppressing the transcription of FOXO3a gene [11]. FOXO3a is phosphorylated, rendering it inactive, and is transported to the cytoplasm from nucleus via 14-3-3 chaperones [12]. In case of inactivation of Akt, FOXO3a transcribes to produce p53, PTEN, Bim-1, FasL, GaDD45, and cyclin G2 promoting cell death or cell cycle arrest [12]. Polymorphism in the FOXO3a gene leads to loss of control over the cell cycle leading to lymphoproliferation which results in formation of tumors and cancers such as prostate cancer and acute lymphoblastic leukemia [13, 14].

FOXO3a had been reported to have redundant roles in suppressing inflammatory cytokine production by dendritic cells and initiation of TGF*β*-1 dependent pathway in monocytes [15, 16]. Through TGF*β*-1 pathway, FOXO3a reduces production of proinflammatory cytokines including TNF-*α*, IL-4, and IL-13 and increases production of anti-inflammatory cytokine IL-1 [17–19]. Rheumatoid Arthritis studies revealed that phosphorylation of FOXO3a gene took place in lymphocytes, monocytes, and macrophages implying FOXO3a's role in inflammatory cell activation [20]. FOXO3a deficient mice models when triggered for cell proliferation exhibited lymphoproliferation, inflammation of airways, salivary glands, lungs, and a remarkable increase in activity of helper T cells [20]. Survival of neutrophils, mast cells, and macrophages has also been associated with FOXO3a [2, 3].

Polymorphism studies of FOXO3a have also proven association of FOXO3a and inflammatory diseases as chronic obstructive pulmonary disease, Rheumatoid Arthritis, Crohn's disease, and inflammatory bowel's disease [21–23]. Thus based on these studies we hypothesized that the hyperactivity of T cells, neutrophils, and mast cells, increased production of proinflammatory cytokines, and downregulation of anti-inflammatory cytokines in asthma patients may be linked to the polymorphism of FOXO3a gene.

## 2. Materials and Methods

### 2.1. Study Design

The study design was approved by the Institutional Ethics Committee of Tilak Ayurved Mahavidyalaya (TAMV) and Seth Tarachand Ramnath Charitable Ayurvedic Hospital (IEC Approval number RSTH/RES/IEC/429/2011). Written informed consent was obtained from all the patients and healthy control subjects before recruitment. Blood was obtained from a total of 114 asthma patients and 142 healthy controls. Healthy subjects were nonsmokers and had no history of asthma, allergy, or other diseases. Demographic and baseline clinical characteristics of recruited subjects are summarized in Table 1.

Diagnosis of bronchial asthma was done by a chest physician with the help of diagnostic criteria as per ICD-9-CM classifications [24]. Spirometry was performed according to 2005 ATS/ERS norms to measure FEV1 and FVC.

### 2.2. Blood Collection and DNA Isolation

Two mL of venous blood was collected in an EDTA coated BD vacutainer (Becton, Dickinson Company Franklin Lakes, NJ, USA) from healthy controls and asthmatic patients. Genomic DNA was isolated and purified from the blood samples using QIAamp DNA Mini Kit (Qiagen, Hilden, Germany) in accordance with the manufacturer's instructions and was stored at −80°C till further use. Isolated DNA was analyzed on 0.8% agarose gel. The purity and yield (ng) of isolated DNA were determined using NanoDrop spectrophotometer and analyzed using ND1000 software (NanoDrop Technologies, Wilmington, USA). The DNA samples used for PCR-RFLP had a purity ratio (260/280) between 1.7 and 2.

### 2.3. Primer Designing

The primers were designed using Gene Runner software (Version 3.05). Details of the primers are given in Table 2.

### 2.4. PCR Amplification of Genomic DNA

The extracted genomic DNA was amplified using Polymerase Chain Reaction (PCR). The primers used for amplification of FOXO3a gene are shown in Table 2 and were synthesized (Eurofins Genomics, Bangalore, India). PCR was performed on Veriti PCR (Applied Biosystems, USA). The PCR reaction comprised 20 *μ*L volume with 0.5 *μ*L of 100 ng DNA, 1 *μ*L of 10 mM dNTPs (Bangalore Genei, India), 0.3 *μ*L of 10 pmol of forward and reverse primer each, 0.3 *μ*L of 3 U* Taq* DNA polymerase (Bangalore Genei, India), 3*μ*L of* Taq* polymerase Buffer A (Bangalore Genei, India), and 14.6 *μ*L of deionized water. The thermal cycling conditions were 5 minutes for initial denaturation at 95°C, 35 cycles at 95°C for 30 seconds for denaturation, 30 seconds at 62°C for annealing, and 1 minute at 72°C for extension, followed by 5 minutes at 72°C for final extension. The PCR products were separated on a 1.5% agarose gel containing 1.5 *μ*L (stock of 10 mg/mL) ethidium bromide. A 100 bp DNA marker (Bangalore Genei, India) was used as a size standard for each gel lane. The gel was visualized under UV light using a gel documentation unit (Alpha Imager HP).

### 2.5. Restricted Fragment Length Polymorphism (RFLP) Analysis

**Figure 1:**
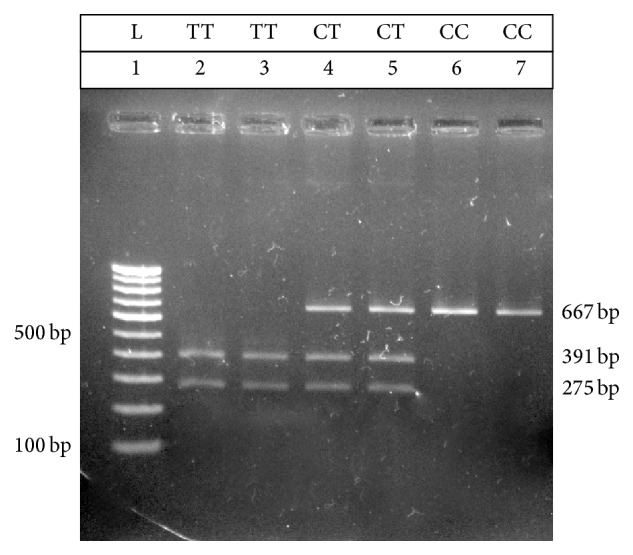
Gel representing typical restriction fragment length polymorphism patterns obtained by subjecting PCR products to restriction digestion by enzyme,* PagI* (New England Biolabs Inc., USA). Lane 1 consists of the 100 bp DNA marker (Bangalore Genei, India). Lanes 2 and 3 represent RFLP pattern for homozygous mutant alleles (TT). Lanes 4 and 5 represent RFLP pattern for heterozygous (CT) alleles. Lanes 6 and 7 represent RFLP pattern for homozygous wild type (CC) alleles.

PCR products are subjected to restriction digestion by the enzyme,* PagI* (New England Biolabs Inc., USA). The RFLP reaction is a 30 *μ*L mixture which comprises 10 *μ*L of PCR product, 14 *μ*L of nuclease-free water, 5 *μ*L of 10x NE Buffer (New England Biolabs Inc., USA), and 1 *μ*L of restriction enzyme,* PagI* (New England Biolabs Inc., USA). The reaction mixture is incubated at 37°C for 15 minutes and the fragments were separated on a 2% agarose gel containing 2 *μ*L (stock of 10 mg/mL) ethidium bromide (Table 3 and Figure 1). The restricted fragments were analyzed for determination of genotype frequencies.

### 2.6. Statistical Analysis

The data were analyzed using the GraphPad PRISM statistical software (version 5.0, San Diego, CA, USA). The statistical difference between genotype and allelic frequencies in asthmatics and controls was calculated using Chi-square (*χ* ^2^) test. Microsoft Excel Sheet was used to analyze whether the distribution of genotypes was in agreement with Hardy-Weinberg equilibrium. Further, odds ratio with 95% confidence interval (CI) was calculated to determine the association between asthmatic patients and healthy control's genotypic and allele frequencies. Odds ratio was also calculated after stratification based on gender. Odds ratio was used to statistically demonstrate a positive dose-response relationship of T allele (mutant allele) with asthma.

## 3. Results

### 3.1. Asthma Patients

Genotype frequencies of 114 asthma patients were studied using PCR-RFLP to detect the presence of single nucleotide polymorphism of FOXO3a gene (rs13217795). rs13217795 single nucleotide polymorphism is characterized by C>T. The genotype frequency of mutant type (TT) had the highest frequency at 51.75% and genotype, and homozygous wild type (CC) had the lowest frequency at 9.65% (Table 4). The asthma group fits in the Hardy-Weinberg equilibrium and has a* p* value of 0.509. The allelic frequencies were calculated using genotype frequencies which indicated that the mutant allele (T) had a higher frequency (71.05%) than the wild allele (C) (28.95%) in asthmatic patients (Table 4). The asthma patient population was further stratified by gender and it was observed that males had a higher heterozygous wild type (CT) frequency (47.82%) than females (24.44%) and females had a higher mutant type (TT) frequency (39.13%) than males (71.11%) (Table 5).

### 3.2. Healthy Controls

Genotype frequencies of 142 healthy controls were studied using PCR-RFLP to detect the presence of single nucleotide polymorphism of FOXO3a gene (rs13217795). The genotype frequency of homozygous wild type (CC) had the highest frequency at 50.70% and genotype frequency of mutant type (TT) had the lowest frequency at 12.67% (Table 4). The healthy controls fit in the Hardy-Weinberg equilibrium and have a* p* value of 0.0866. Allelic frequencies were calculated which indicated that wild allele (C) had a higher frequency (69.01%) than mutant allele (T) (30.99%) in healthy controls (Table 4). The healthy control population was stratified by gender and it was observed that homozygous wild type (CC), heterozygous wild type (CT), and mutant type (TT) frequencies are almost similar to each other (Table 5).

### 3.3. Association Studies

Chi-square test indicated significant association between the asthma and control groups (*p* < 0.0001) for both genotype and allele frequencies. The odds ratio for genotype frequency (Table 4) was calculated keeping CC (wild type) as a baseline. Odds ratio for CT (heterozygous wild type) was 5.54 with 95% confidence interval (2.48 to 12.62) and TT (mutant) was 21.45 with 95% confidence interval (8.78 to 53.84). Odds ratio for allelic frequencies (Table 4) was also significant, 5.47 for 95% confidence interval (3.67 to 8.16).

As per genotype frequency analyzed data, asthmatics are 5.54 times more likely to have a CT allele as compared to CC and 21.45 times more likely to have a TT allele. Both these associations are statistically significant because both the limits of confidence intervals are above 1.0. Positive correlation indicates that people with CT allele have 5.54 times higher risk of developing asthma and those with TT allele have 21.45 times higher risk of developing asthma as compared to people with CC allele. If TT allele can be considered as a higher exposure to T as compared to CT, then the above statistics demonstrate a positive dose-response relationship of T allele with asthma.

After stratification of asthma and healthy control population based on gender, the odds ratio and 95% confidence interval were calculated separately from genotype and allele frequency which indicated that “T” allele is a risk factor for asthma in both the genders but its effect is more pronounced in females (OR = 56.88; 95% CI = 11.386 to 284.219) as compared to males (OR = 13.33, 95% CI = 4.689 to 37.911). Odds ratio from allelic frequency also indicated that females (OR = 10.6098; 95% CI = 5.4442 to 20.67) are at a higher risk of asthma than males (OR = 3.9562; 95% CI = 2.432 to 6.433) (Table 5).

## 4. Discussion

Asthma affects more than a third of Indian population; thus the need to unravel the main reason for its prevalence is of utmost importance [1]. Asthma may occur due to genetic or environmental factors such as air pollution. The onset of asthma may occur at any time in life, as early as birth to as late as old age. Asthma is an inflammatory disorder characterized by heightened immunological response by immunological cells thereby disrupting immunoregulation.

More than 100 genes and their SNP have been studied and are found to have strong association with asthma in the Indian population [1]. This study mainly focuses on the possible association of FOXO3a and asthma. FOXO3a is associated with a number of inflammatory diseases such Crohn's disease, Rheumatoid Arthritis synovial tissue, and Sjögren's syndrome [22, 23]. FOXO3a regulates activities such as production of anti-inflammatory cytokine, IL-10, NF-*κ*B, IFN-*α*, and IL-2 also maintaining excessive proliferation of T cells, neutrophils, and macrophages thus playing an important role in inflammatory pathways [3, 18, 20].

FOXO3a is responsible for differentiation and maintenance of T cells. Rheumatoid Arthritis studies revealed that phosphorylation of FOXO3a took place in lymphocytes and when FOXO3a deficient mice were stimulated for proliferation, spontaneous lymphoproliferation was observed along with inflammation of organs such as salivary glands, lungs, and increased activity of helper T cells, supporting an important role for FOXO3a in preventing T cell hyperactivity which is one of the premier causes of asthmatic inflammation [20].

FOXO3a works in concert with FasL signalling to maintain the proliferation of T cells in case of inflammatory activity [20]. FOXO3a induces mitochondrial apoptosis via FasL but in its absence, proliferation of helper T cells suffers no restriction thereby resulting in hyperactivity of T cells resulting in activation of related cytokines [20].

FOXO3a also appears to regulate neutrophilic activity and survival by suppressing the transcription of FasL and proapoptotic proteins Bim and Bak [25]. Studies indicate that mutation in the FOXO3a gene has resulted in increased survival of neutrophils in Rheumatoid Arthritis samples compared to control [3]. As neutrophils are one of the main components when it comes to eliciting an immune response, their survival and maintenance are of crucial importance in asthmatic inflammation [3]. Systemic lupus erythematosus animal models suggest that upregulation of FOXO3a blocks NF-*κ*B activation and IFN-*γ* secretion thereby preventing inflammatory activation and cellular apoptosis [26]. Loss of inhibition of NF-*κ*B by FOXO3a leads to release of autoreactive T cells, surmounting an immune response.

IL-2 and IFN-*γ* genes are proinflammatory targets for FOXO3a gene [26, 27]. In case of an immunogenic response the secretion of IFN-*γ* and IL-2 is drastically increased which acts as a trigger for the corresponding cytokines [26, 27]. Single nucleotide polymorphism of FOXO3a may result in upregulation of these genes thus promoting inflammation.

Cytokine, TNF-*α*, has been proved to be a major contributor in intestinal inflammation [23]. TNF-*α* along with upregulation of IL-8 results in downregulation of FOXO3a resulting in excessive inflammation by stimulating NF-*κ*B [23].

Mast cells play an enormous role in inflammation of smooth muscle in asthma [2]. FOXO3a, like neutrophils, maintained the survival of mast cells with the help of proapoptotic protein Bim [3, 28]. Thus inactivation of FOXO3a due to phosphorylation or mutation would amount to increased expression of mast cells.

Our study for the first time shows that FOXO3a SNP (rs13217795) is significantly associated with Indian asthmatics plausibly contributing to the hyperactivity of T cells, neutrophils, and mast cells, increased production of proinflammatory cytokines, and downregulation of anti-inflammatory cytokines. In addition to the highly significant association of FOXO3a and asthma, gender based stratification also indicated that the mutant “T” allele has a much more pronounced risk rate of asthma in females than males.

Our study involves a smaller sample size and the population is restricted to the Maharashtra state of India. Thus population based study with larger sample size needs to be carried out.

## 5. Conclusions

Genotype frequencies of 114 asthma patients and 142 healthy controls were studied using PCR-RFLP technique to detect the presence of single nucleotide polymorphism of FOXO3a gene (rs13217795). Both the groups fit the Hardy-Weinberg equilibrium. The Chi-square test indicated significant positive relationship between asthma and CT and TT genotypes (*p* < 0.0001).

## Figures and Tables

**Table 1:** Demographic and clinical characteristics of asthma patients and healthy controls.

Category	Total	Male	Female	Mean age	FEV1^a^	FVC^b^
				(years)	(% predicted)	(% predicted)
Asthma group	114	69	45	41.8 ± 8.7	64.9 ± 12.7	71.3 ± 2.3
Control group	142	78	64	42.9 ± 11.5	93.6 ± 5.4	95.0± 4.3

^a^Forced expiratory volume in 1 second, ^b^forced vital capacity.

**Table 2:** Technical data for analysis of SNP in the human FOXO3a gene.

Gene name	SNP	Primers	GC content (%)	Annealing temperature (°C)	Restriction enzyme^*∗*^	PCR product size (bp)
FOXO3a	rs13217795	Forward: 5′-CTCCTTGGTCAGTTTGGTG-3′ Reverse: 5′-ATGAGTGAAGATGGAAGTAAGC-3′	52.6% 40.9%	62°C	*PagI*	667 bp

^*∗*^New England Biolabs Inc., USA.

**Table 3:** Analysis of RFLP fragments.

Type	Fragment sizes
Homozygous wild type (CC)	667 bp
Heterozygous wild type (CT)	667 bp, 321 bp, and 275 bp
Mutant type (TT)	321 bp and 275 bp

**Table 4:** The genotype and allele frequencies for asthmatic patients and control subjects of rs13217795 of FOXO3a gene.

		Frequencies		*p* value^*∗*^	Degrees of freedom
		Asthmatic patients	Healthy controls		
		(*n* = 114)	(*n* = 142)		
Genotype	CC	11 (9.65%)	72 (50.70%)	<0.0001	2
	CT	44 (38.6%)^a^	52 (36.61%)^a^		
	TT	59 (51.75%)^b^	18(12.67%)^b^		

Allele	C	66 (28.95%)^c^	196 (69.01%)^c^	<0.0001	1
	T	162 (71.05%)^c^	88 (30.99%)^c^		

^*∗*^Chi-square test (*χ* ^2^).

*Odds ratio from genotypic frequency (keeping CC as baseline)*:

^a^CT—5.54; 95% confidence interval = 2.48 to 12.62.

^b^TT—21.45; 95% confidence interval = 8.78 to 53.84.

*From allelic frequency*:

^c^odds ratio = 5.47; 95% confidence interval = 3.67 to 8.16.

**Table 5:** Distribution of the rs13217795, C>T polymorphism according to gender in the study groups.

Gender		Males		Females	
		Asthmatic patients (*n* = 69)	Healthy controls (*n* = 78)	Asthmatic patients (*n* = 45)	Healthy controls (*n* = 64)
Genotype	CC	9 (13.04%)	40 (51.28%)	2 (4.44%)	32 (50%)
	CT	33 (47.82%)^a^	29 (37.17%)^a^	11 (24.44%)^c^	23 (35.93%)^c^
	TT	27 (39.13%)^b^	9 (11.54%)^b^	32 (71.11%)^d^	9 (14.06%)^d^

Allele	C	51 (36.95%)^e^	109 (69.87%)^e^	15 (16.66%)^f^	87 (67.96%)^f^
	T	87 (63.04%)^e^	47 (30.12%)^e^	75 (83.33%)^f^	41 (32.03%)^f^

*Odds ratio from genotypic frequency*:

Males: odds ratio keeping CC as baseline:

^a^CT—5.057; 95% confidence interval = 2.10 to 12.174.

^b^TT—13.33; 95% confidence interval = 4.689 to 37.911.

Females: odds ratio keeping CC as baseline:

^c^CT—7.652; 95% confidence interval = 1.5463 to 37.867.

^d^TT—56.88; 95% confidence interval = 11.386 to 284.219.

*Odds ratio from allelic frequency*:

^e^Males: odds ratio = 3.9562; 95% confidence interval = 2.432 to 6.433.

^f^Females: odds ratio = 10.6098; 95% confidence interval = 5.4442 to 20.67.
